# SCARAP: scalable cross-species comparative genomics of prokaryotes

**DOI:** 10.1093/bioinformatics/btae735

**Published:** 2024-12-11

**Authors:** Stijn Wittouck, Tom Eilers, Vera van Noort, Sarah Lebeer

**Affiliations:** Lab of Applied Microbiology and Biotechnology, Department of Bioscience Engineering, University of Antwerp, Antwerpen 2020, Belgium; Lab of Applied Microbiology and Biotechnology, Department of Bioscience Engineering, University of Antwerp, Antwerpen 2020, Belgium; Faculty of Bioscience Engineering, KU Leuven, Leuven 3001, Belgium; Institute of Biology Leiden, Leiden University, Leiden 2333 BE, The Netherlands; Lab of Applied Microbiology and Biotechnology, Department of Bioscience Engineering, University of Antwerp, Antwerpen 2020, Belgium

## Abstract

**Motivation:**

Much of prokaryotic comparative genomics currently relies on two critical computational tasks: pangenome inference and core genome inference. Pangenome inference involves clustering genes from a set of genomes into gene families, enabling genome-wide association studies and evolutionary history analysis. The core genome represents gene families present in nearly all genomes and is required to infer a high-quality phylogeny. For species-level datasets, fast pangenome inference tools have been developed. However, tools applicable to more diverse datasets are currently slow and scale poorly.

**Results:**

Here, we introduce SCARAP, a program containing three modules for comparative genomics analyses: a fast and scalable pangenome inference module, a direct core genome inference module, and a module for subsampling representative genomes. When benchmarked against existing tools, the SCARAP pan module proved up to an order of magnitude faster with comparable accuracy. The core module was validated by comparing its result against a core genome extracted from a full pangenome. The sample module demonstrated the rapid sampling of genomes with decreasing novelty. Applied to a dataset of over 31 000 *Lactobacillales* genomes, SCARAP showcased its ability to derive a representative pangenome. Finally, we applied the novel concept of gene fixation frequency to this pangenome, showing that *Lactobacillales* genes that are prevalent but rarely fixate in species often encode bacteriophage functions.

**Availability and implementation:**

The SCARAP toolkit is publicly available at https://github.com/swittouck/scarap.

## 1 Introduction

Comparative genomics helps us understand the functional diversity of prokaryotes. In addition, it allows us to reconstruct evolutionary history as well as the evolutionary process. Two computational tasks that are central to prokaryotic comparative genomics are pangenome inference and core genome inference. Pangenome inference entails the clustering of all (predicted) genes in a set of genomes into gene families, or orthogroups, with genes from the same gene family assumed to share a common function. The pangenome can be used to perform prokaryotic genome-wide association studies (GWAS), which examine whether one or more genes are associated with a phenotype or some other condition of interest (Falush 2016). The pangenome can also be used to infer functional associations between gene families (Whelan *et al.* 2020). Furthermore, the pangenome allows for the comprehensive study of the evolutionary history of a set of sequenced prokaryotic strains. For example, it can be used to infer a phylogenetic tree of the strains and to model gene gain and loss and horizontal transfers (Tria and Martin 2021). Finally, the pangenome can be used to calculate pairwise average nucleotide identity (ANI) values for a set of genomes since it informs us on which sequences are homologous and can therefore be compared.

Historically, pangenome inference has usually been implemented as a three-step process (Enright *et al.* 2002): (i) an all-versus-all comparison of all (predicted) genes in the genomes, (ii) the transformation of the resulting sequence comparison scores to correct for phylogenetic distance and other factors, and (iii) the application of a clustering algorithm on the transformed scores to obtain gene families. Because the all-versus-all gene comparison step scales quadratically with the total number of genes, this approach is very slow for larger datasets. For species-level datasets (i.e. sets of genomes belonging to the same species), faster and more scalable approaches have been developed (Page *et al.* 2015, Tonkin-Hill *et al.* 2020). However, these tools are not applicable to genus-level datasets and higher taxonomic ranks.

The core genome is the fraction of the pangenome consisting of all gene families that are present in (almost) all genomes in the dataset in at least one copy. For some tasks in comparative genomics, the core genome is sufficient as input and the full pangenome is not needed; examples are the inference of a high-quality phylogeny (Parks *et al.* 2018) or population structure (Raj *et al.* 2014). In addition, the core genome can be leveraged for quality control of the genomes and more specifically, to calculate estimates of their completeness and contamination (Parks *et al.* 2015). It can also be used to calculate pairwise ANI values based on core genes only (cANI values), which has the advantage over traditional ANI values that all genome comparisons are based on the same set of genes (Wittouck *et al.* 2019, Zheng *et al.* 2020).

A core genome is usually determined in one of two approaches: either by inferring the full pangenome and then subsetting it to the core genes only (Coleman *et al.* 2021, de la Haba *et al.* 2023), or by searching the genomes with a set of sequences or profiles of pre-determined core genes (Parks *et al.* 2018, Asnicar *et al.* 2020). Obviously, the former strategy suffers from the same scalability issues as full pangenome inference. The latter strategy is efficient, but it requires that core genes are known in advance and does not guarantee that the set of pre-determined core genes is complete. A tool for direct and *de novo* inference of the core genome is currently lacking.

A straightforward strategy to save computation time in comparative genomics analyses, is to apply the pipeline of interest to a representative subset of the genome dataset. While fast tools exist to dereplicate a genome dataset given a maximum genome similarity cutoff (Olm *et al.* 2017), a scalable approach for subsampling a given number of representative genomes does not exist yet, to the best of our knowledge. A way to perform such subsampling with currently available tools, would be to calculate a pairwise ANI matrix for the genomes, apply a clustering technique, and select one genome per cluster. The issue with this approach is that it again scales quadratically with the number of input genomes, due to the pairwise ANI calculation step. In addition to speeding up downstream analyses, representative subsampling can also help to reduce sampling bias in genome datasets, where particular species, lineages or even strains are often overrepresented.

In this work, we describe three tools aimed at enabling faster and more scalable comparative genomics of Bacteria and Archaea: first, a tool for fast and scalable full pangenome inference; second, a tool for direct and database-free core genome inference; and third, a tool for selecting a representative subset from a larger set of genomes. All tools work on datasets containing genomes from the same or from different species. The tools have been implemented in the SCARAP toolkit, which is publicly available at https://github.com/swittouck/scarap.

## 2 Materials and methods

### 2.1 The SCARAP pan module

Given a set of proteomes, the SCARAP pan module infers a pangenome in two steps: a fast clustering of all amino acid sequences into ‘superclusters,’ followed by the iterative binary splitting of each supercluster into orthogroups. The superclusters are created through two consecutive clustering operations using MMseqs2 (Steinegger and Söding 2017, 2018). The first clustering is the ‘cascaded clustering’ procedure of MMseqs, as implemented in its cluster module. This results in what we call ‘preclusters.’ A second, profile-based clustering step is then added for increased sensitivity. First, an alignment profile and a consensus sequence are created for each precluster. These are then compared in an all-versus-all fashion. Based on this comparison, the preclusters are clustered into superclusters.

The iterative binary splitting of a supercluster into orthogroups happens as follows. Each iteration starts with the selection of N (default: 32) representative sequences through our FICLIN algorithm (see Supplementary Text S1). If the number of sequences is smaller than M (default: 512), all sequences are selected as representatives. Next, the representatives are aligned with MAFFT, a distance matrix is created from the alignment, and average-linkage hierarchical clustering is performed. The two top-level clusters are then determined and re-inflated with the full set of sequences by assigning sequences to the cluster of their closest representative. Finally, an assessment is made of the proposed binary split of the cluster. If the two subclusters are present in the same genome at least as often as under a null model where the division into subclusters is random, the split is accepted and the splitting process continues for each of the subclusters. If the two subclusters are less often present in the same genome than expected under the null model, the split is rejected and the cluster becomes a single orthogroup (see Supplementary Text S1 for a detailed description of the pan module).

### 2.2 The SCARAP core module

The SCARAP core module infers a core genome for a set of proteomes by first inferring the full pangenome of a random set of “seed genomes”. Specifically, the module follows four steps. The first step is the selection of a random subset of 100 seed genomes and the inference of their full pangenome using the pan module. The second step consists of the identification of candidate core genes as all gene families present in a single copy in at least 90% of the seed genomes, the creation of a multiple sequence alignment of these candidate core genes with MAFFT (Katoh and Standley 2013) and their conversion to alignment profiles with MMseqs2. In the third step, the profiles are searched against all seed genome amino acid sequences with MMseqs search. A score cutoff is then determined per profile as the mean of the scores of sequences belonging to the candidate core gene family and the scores of sequences not belonging to the gene family. The score cutoffs are then applied to re-determine which seed sequences belong to the candidate core genes. The final core genes are then selected as the candidate core genes with a single-copy presence in at least 95% of seed genomes. In the fourth and final step, the core gene profiles are searched against the non-seed genomes and the profile-specific score cutoffs are applied to identify their member sequences in these genomes.

The default values for the number of seed genomes, the minimum prevalence of candidate core genes, and the minimum prevalence of final core genes can all be changed by the user. In addition, an optional maximum number of final core genes can be requested.

### 2.3 The SCARAP sample module

Given a pre-identified core or pangenome for a set of input proteomes, the SCARAP sample module samples a requested number of genomes in a representative manner. Alternatively, the module can sample genomes until a requested maximum sequence identity between sampled genomes is reached. The sampling algorithm used is our FICLIN algorithm; the same algorithm that is used to select representative sequences during the splitting stage of the SCARAP pan module (see Supplementary Text S1). Instead of using the sequence identity between individual sequences, as in the splitting stage of SCARAP pan, the algorithm here uses the ANI value between genomes, where different types of ANI values can be calculated. When the full pangenome of the genomes is supplied, the ANI between two genomes is calculated as the mean of the per-gene sequence identity values for all genes with a single copy in both the first and second genomes. When only the core genome is supplied, the resulting ANI will be a core-genome-ANI (cANI). Optionally, extreme per-gene identity values can be purged before calculating the mean. For example, setting the method argument to ‘mean90’ will use only the middle 90% of per-gene sequence identity values to calculate the ANI.

### 2.4 Benchmarking of SCARAP

Six different datasets were used to benchmark and demonstrate the SCARAP pan, core, and sample modules. The SimPan dataset consisted of 100 genomes simulated *de novo* with the SimPan tool (Brown *et al.* 2016, Zhou *et al.* 2020) using an nucleotide identity setting of 90% to represent a genus-level dataset. The Tonkin-Hill dataset was taken from Tonkin-Hill *et al.* (2020); it was simulated using an *Escherichia coli* genome as a starting point and represents a sub-species-level population. The third dataset consisted of one representative genome per genus of the order *Lactobacillales* and was compiled as follows. From all public *Lactobacillales* genomes identified by the Genome Taxonomy Database (GTDB; Parks *et al.* 2018), release 207, the best-quality genome was selected for each genus. Here, quality was calculated as the estimated completeness minus estimated contamination value, as precalculated with the tool checkM (Parks *et al.* 2015) by the GTDB. These genomes were downloaded from GenBank (Sayers *et al.* 2022) and gene prediction and translation were performed with Prodigal (Hyatt *et al.* 2010).

The fourth dataset consisted of all publicly available genomes of the genus *Lactiplantibacillus*, again as defined by release 207 of the GTDB. Gene prediction was again performed with Prodigal. The fifth and sixth datasets were the OrthoBench dataset with 70 curated orthogroups from 12 eukaryotic genomes (Trachana *et al.* 2011, Emms and Kelly 2020), and the paraBench dataset with 52 curated orthogroups from 17 eukaryotic genomes (Derelle *et al.* 2020).

The SCARAP pan module was benchmarked against four highly regarded pangenome tools: OrthoFinder (Emms and Kelly 2015, 2019), SonicParanoid (Cosentino and Iwasaki 2019), Broccoli (Derelle *et al.* 2020), and PIRATE (Bayliss *et al.* 2019). OrthoFinder was tested both with BLAST and MMseqs2 as sequence search engines, while SonicParanoid was tested in its ‘fast’ and ‘sensitive’ modes. PIRATE can only be run on prokaryotic datasets so it was not applied to the OrthoBench and paraBench datasets. Tools were compared based on their speed and accuracy (precision, recall, and *F*-measure) on the SimPan, Tonkin-Hill, OrthoBench, and paraBench datasets and on their speed on the *Lactobacillales* dataset.

The SCARAP core module is meant to be a shortcut to the strategy of inferring the full pangenome and then subsetting it to core orthogroups only using an orthogroup prevalence threshold. Therefore, to assess the accuracy of the core module, we first ran the SCARAP pan module on the *Lactiplantibacillus* dataset. We then determined the reference core genome by applying a minimum single-copy prevalence of 95%. Next, we ran the SCARAP core module on the same dataset with the same threshold of 95% minimum single-copy prevalence. Finally, we compared this result against the reference core genome by calculating the precision, recall, and F-measure.

To demonstrate the SCARAP sample module, we ran it on the *Lactiplantibacillus* dataset using four types of ANI values: (i) the core genome ANI (cANI) using all core genes identified by the SCARAP core module, (ii) the cANI using a subset of 100 core genes, (iii) a trimmed cANI (tcANI) where the 5% genes with the lowest and highest sequence identity values for each genome comparison were removed, and (iv) the tcANI but using only 100 core genes. To assess the effect of using only 100 core genes, we calculated the Spearman correlations of cANI or tcANI values between the full core genome and the subset of 100 core genes.

We also explored the SCARAP genome sampling of the *Lactiplantibacillus* dataset in phylogenetic terms. First, we used the subset of 100 core orthogroups to create a nucleotide ‘supermatrix’ with the SCARAP concat module. This module removes all gene copies from genomes with more than one copy of a core gene, aligns the core genes on the amino acid level with MAFFT, replaces the amino acid sequences with the corresponding nucleotide sequences in the alignments, and concatenates the nucleotide alignments together horizontally. We then inferred a maximum likelihood phylogeny with IQ-TREE (Nguyen *et al.* 2015), with the general time reversible (GTR) nucleotide substitution model and with among-site rate heterogeneity modelled as a discretized gamma distribution with four rate categories (G4). We then explored the first ten genomes sampled using tcANI distances based on 100 core genes by inferring ‘ancestor discoveries.’ Each sampled genome implies the discovery of the most recent common ancestor of the sampled genome and the most closely related genome already sampled. Intuitively, a good sampling process discovers older ancestors before more recent ones.

### 2.5 Creation of a pangenome database for *Lactobacillales*

To demonstrate how the SCARAP modules can be combined to gain novel insights from a large-scale genome dataset, we inferred and explored a pangenome for the order *Lactobacillales*. First, all publicly available genomes of *Lactobacillales* were identified from release 207 of the GTDB. We noticed that some species had received an sp.-identifier from the GTDB (e.g. *Streptococcus* sp001937065) but had validly published species names (e.g. *Streptococcus caviae*); we replaced these sp-identifiers with the published species names by cross-referencing the strain names of these genomes with the LPSN list of type strains, version 2023-03-23 (Euzéby 1997, Parte *et al.* 2020). Next, we downloaded all genomes from GenBank and performed gene prediction with Prodigal.

We then aimed to representatively subset these genomes. We first selected the GTDB representative genome from each species and inferred their pangenome with SCARAP pan. Next, we created multiple sequence alignments for 100 (largely) single-copy core genes from this pangenome with the SCARAP build module and identified these genes in all genomes with the SCARAP search module. We then used these core genes to run the SCARAP sample module on each species, for all genomes with an overall quality score of at least 90%. We sampled genomes in such a way that (i) all sampled genomes had a tcANI of maximum 99.99% to each other and (ii) maximum 100 genomes were sampled per species.

Finally, we inferred the full pangenome of the representative genomes with SCARAP pan. To estimate species-level core genes, we performed a joint maximum-likelihood optimization of orthogroup prevalence values and genome completeness values; see Supplementary Text S2.

### 2.6 Availability of code and data

SCARAP is available at https://github.com/swittouck/scarap. The SCARAP benchmarking code and results are available at https://github.com/swittouck/benchmark-scarap. The code and data of the *Lactobacillales* representative pangenome are available at https://github.com/swittouck/legen. Finally, the code for the creation of figures and tables in this work is available at https://github.com/swittouck/pangenome-toolkit.

An overview of all software used can be found in Table 1.

**Table 1.** Overview of software used.

Software	Version	Reference
*Pangenome tools*		
Broccoli	v1.1	Derelle *et al.* (2020)
OrthoFinder	v2.5.2	Emms and Kelly (2015, 2019)
PIRATE	v1.0.5	Bayliss *et al.* (2019)
SCARAP	v1.0.0	This work
SonicParanoid	v1.3.8	Cosentino and Iwasaki (2019)
*SCARAP dependencies*		
Biopython	v1.79	biopython.org
ETE Toolkit	v3.1.3	Huerta-Cepas *et al.* (2016)
MAFFT	v7.525	Katoh and Standley (2013)
MMseqs2	release15	Steinegger and Söding (2017, 2018)
Numpy	v1.20.2	Harris *et al.* (2020)
Pandas	v1.3.5	Wes McKinney (2010)
		The pandas development team (2020)
Python	v3.7.12	www.python.org
SciPy	v1.7.3	www.scipy.org
*Preparation of genome datasets*		
GenomeTools	v1.6.2	Gremme *et al.* (2013)
Proclasp	v1.0	github.com/swittouck/proclasp
Prodigal	v2.6.3	Hyatt *et al.* (2010)
SimPan	v0.1	Brown *et al.* (2016), Zhou *et al.* (2020)
*Demonstration of SCARAP core module*		
IQ-TREE	v1.6.12	Nguyen *et al.* (2015)
iTol	v7	Letunic and Bork (2021)
*Table processing and visualization*		
ggpubr	v0.4.0	Kassambara (2023)
R	v4.3.3	R Core Team (2024)
tidyverse	v2.0.0	Wickham *et al.* (2019)

## 3 Results and discussion

### 3.1 The SCARAP pan module is fast and accurate

With the goal of making fast pangenome inference possible not only for the species and genus levels but also for more diverse prokaryotic datasets, we designed a computational tool that can handle up to thousands of genomes overnight on a modern computational system. This tool was implemented as the pan module of the SCARAP toolkit. The module was benchmarked against four other popular, user-friendly pangenome tools that are applicable on datasets above the genus level: OrthoFinder (Emms and Kelly 2015, 2019), SonicParanoid (Cosentino and Iwasaki 2019), Brocolli (Derelle *et al.* 2020), and PIRATE (Bayliss *et al.* 2019).

**Figure 1.**
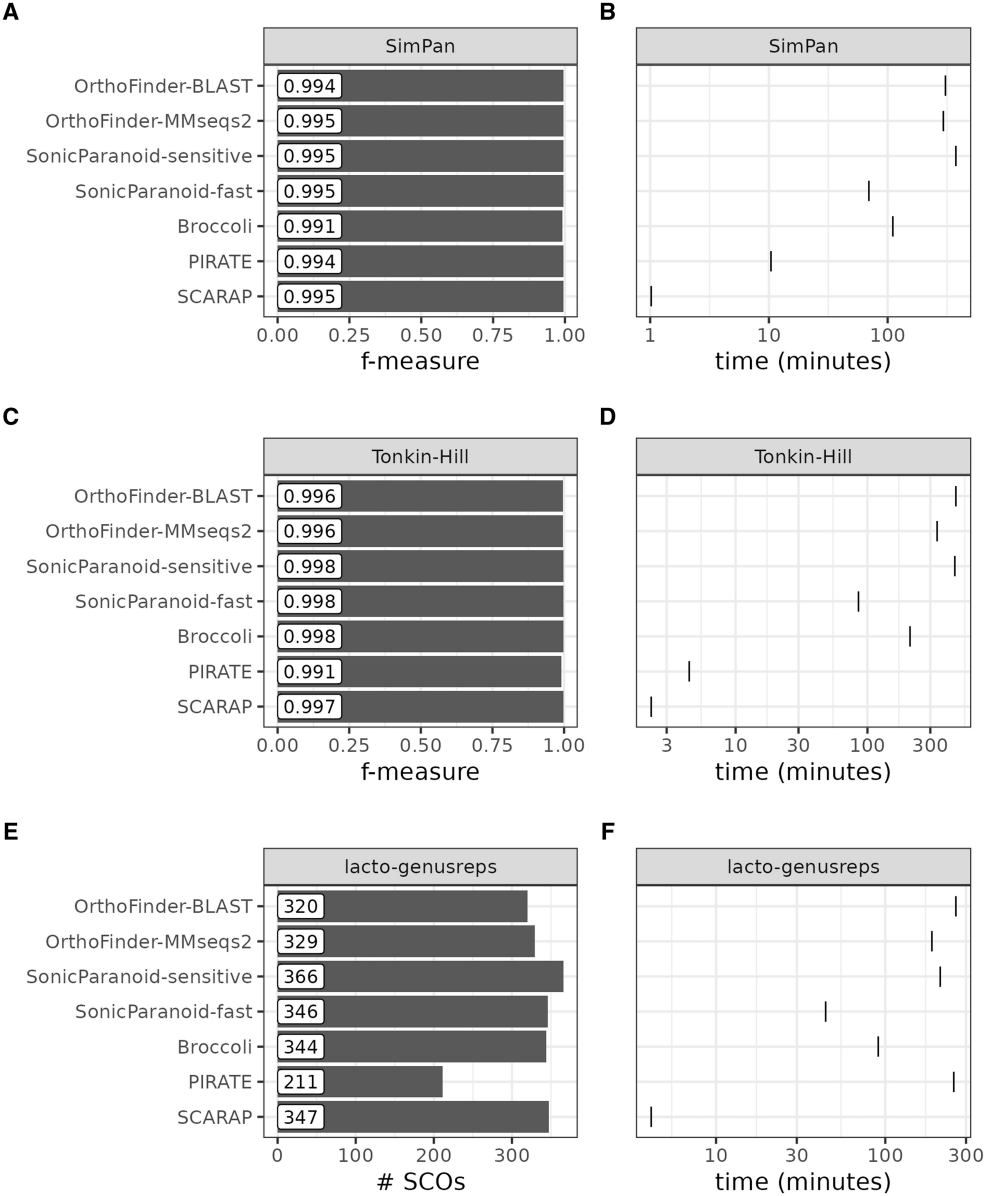
Benchmark of the SCARAP pan module. (A) Accuracy on the SimPan dataset. (B) Speed on the SimPan dataset. (C) Accuracy on the Tonkin-Hill dataset. (D) Speed on the Tonkin-Hill dataset. (E) Accuracy on the lacto-genusreps dataset. Abbreviation: SCO, single-copy core orthogroup. (F) Speed on the lacto-genusreps dataset.

The SCARAP pan parameters were optimized on a dataset with representative genomes of 1,083 *Lactobacillales* species (Supplementary Figs S1 and S2). Based on these optimizations, 32 was chosen as the default number of representative sequences to use in iterative orthogroup splitting and 512 as the default maximum number of sequences to align. When this limit is exceeded, representatives are selected and aligned (see Supplementary Text S1). Next, SCARAP was benchmarked on prokaryotic datasets of moderate size, to allow all tools to finish in reasonable time. All tools performed well on two simulated datasets (Simpan and Tonkin-Hill), with *F*-measures consistently above 99% (Fig. 1A and C). SCARAP was the fastest tool, taking 10 times less time than the second fastest tool (PIRATE) for the SimPan dataset and two times less time on the Tonkin-Hill dataset (Fig. 1B and D). It should be noted that the reported *F*-measures are likely overestimates compared to nonsimulated datasets: the correctness of a pangenome prediction depends not only on the inference process itself but also on upstream steps such as genome sequencing, genome assembly and gene prediction (Marin *et al.* 2024). The values reported here only serve the purpose of comparing different pangenome tools relative to one another.

In order to test SCARAP pan on a nonsimulated, diverse datset, we selected one publicly available genome per genus of the order *Lactobacillales*, for a total of 97 genomes. Lacking a reference pangenome to compare results to, we used the number of recovered single-copy core orthogroups (SCOs) as a rough estimate of pangenome quality. With 347 SCOs, SCARAP performed similarly to most other tools, with only PIRATE standing out on the low end with only 211 SCOs (Fig. 1E). In terms of speed, SCARAP was 10 times faster than the second fastest tool, SonicParanoid in fast mode (Fig. 1F).

To the best of our knowledge, no non-simulated prokaryotic dataset exists with a known reference pangenome. However, two such datasets do exist for eukaryotic genomes. On the OrthoBench dataset (Trachana *et al.* 2011, Emms and Kelly 2020), SCARAP was the fastest but had a slightly lower *F*-measure than the other tools (60% versus 67%–73%, respectively, Supplementary Fig. S3A and B). On the paraBench dataset (Derelle *et al.* 2020), SCARAP achieved the second most accurate result with an *F*-measure of 80%, being tied for speed with SonicParanoid in fast mode (Supplementary Fig. S3C and D).

In conclusion, primarily on prokaryotic datasets, SCARAP pan achieves similar accuracy to alternative pangenome tools while running significantly faster, especially for datasets spanning multiple species.

### 3.2 The SCARAP core module rapidly identifies core genes

We implemented a direct core genome inference strategy, circumventing full pangenome inference, in the core module of SCARAP. To test this module, we ran it on a dataset of 720 genomes of the genus *Lactiplantibacillus*, using the default single-copy core prevalence threshold of 95%. This resulted in the identification of 2093 core genes in 11 min.

To verify the accuracy of the core module, we ran the SCARAP pan module on the same genome dataset, which took 67 min. We next identified core genes in the resulting pangenome by applying the same 95% single-copy prevalence threshold as used by the core module. We then mapped the core orthogroups inferred by both modules to one another: if orthogroups from the two modules shared at least one gene copy in at least 95% of genomes, we considered them ‘the same orthogroup. This mapping allowed us to calculate precision and recall measures of the core module, by considering the core orthogroups identified by the pan module as a reference. The precision and recall values were 91.8% and 95.8%, respectively (Table 2). Thus, the core module proved to be a fast shortcut that can be used to obtain a set of single-copy core genes that is very similar to the one that would be obtained from the pan module.

**Table 2.** Benchmark of the SCARAP core module^a^

SCOs from core module	2093
SCOs from pan module	2006
SCOs from both modules	1921
Precision of core module	91.8%
Recall of core module	95.8%
*F*-measure of core module	93.7%

a Comparison of the SCARAP core module output to the core genome determined by taking the single-copy core genes from the output of the pan module. Abbreviation: SCO, single-copy core orthogroup.

### 3.3 The SCARAP sample module subsamples genomes in linear time

For pangenome inference on the largest datasets, even the SCARAP pan module may take too much time. We therefore also developed the SCARAP sample module: a tool that can rapidly subsample genomes from a larger dataset in a representative manner, given a predetermined set of core genes. The subsampling algorithm we implemented has linear time complexity, as a function of the number of genomes to sample if the total number of genomes remains constant, or vice versa. This means that the sample module will be very fast for small subsample sizes (e.g. sampling 100 representative genomes from a dataset with 10 000 genomes), but slow for subsample sizes that approach the total size of the genome dataset. To test the SCARAP sample module, we ran it on the 720 *Lactiplantibacillus* genomes, using their core genome inferred by the SCARAP core module. This took 3 h and 13 min, or approximately 16 s per sampled genome. It is important to point out here that we sampled all genomes for demonstration purposes but in practical applications, the sample module will be used to sample a given number of genomes. On our dataset, subsampling the first 10 *Lactiplantibacillus* genomes took 160 s.

**Figure 2.**
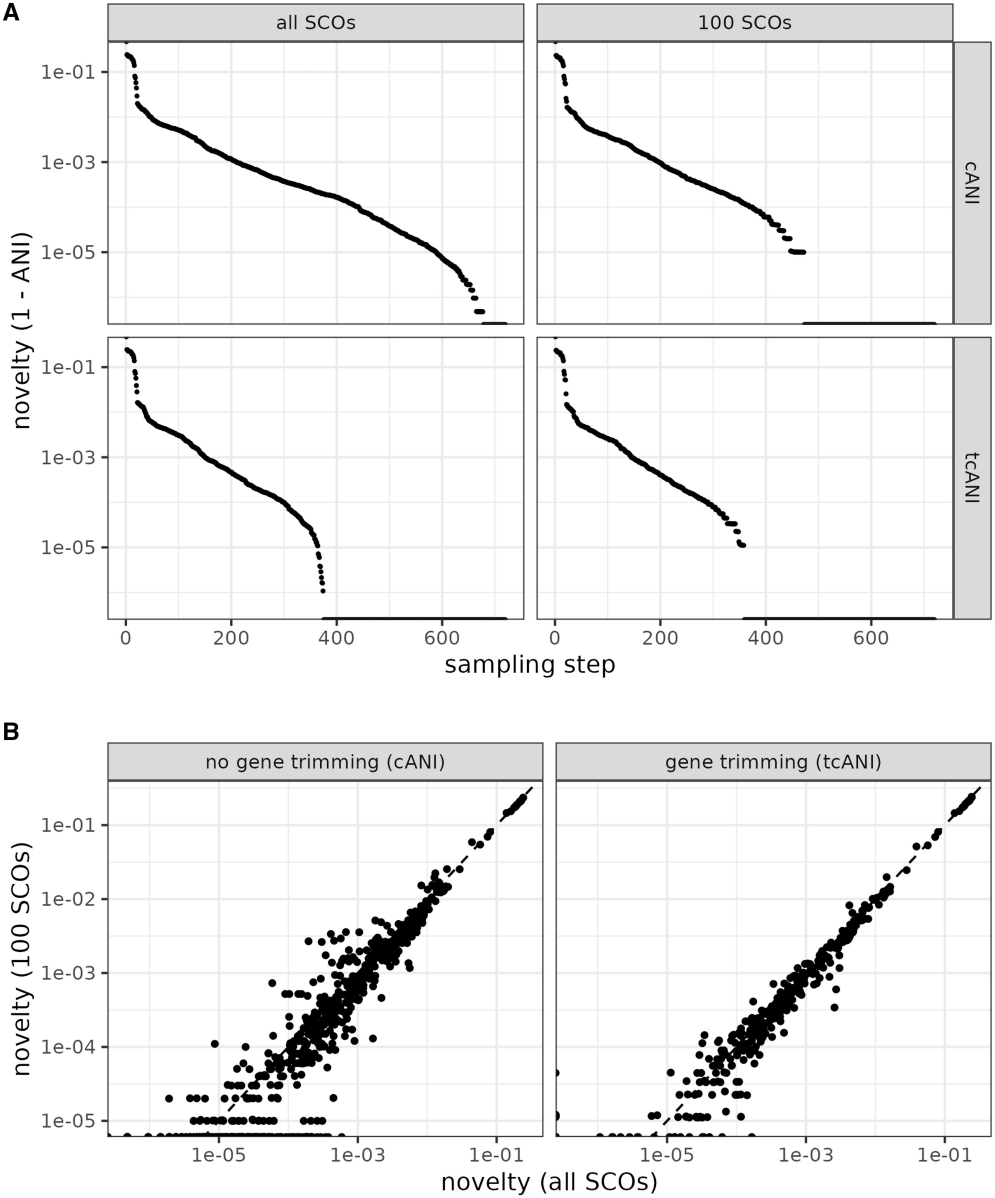
Demonstration of the SCARAP sample module. (A) Novelty curves of sampling genomes using all single-copy core genes (SCOs) versus a subset of 100 SCOs, without trimming genes with the smallest and largest identity values (cANI) and with trimming of such genes (tcANI). (B) Correlation between the cANI calculated from the full core genome and from 100 core genes and the same correlation but for tcANI. In (B), each point represents an independent comparison between two genomes.

To visualize the sampling process, we plotted the ‘novelty curve” of the sampled genomes. We define novelty as the distance (one minus the cANI) of a genome to the closest genome that was already sampled. The higher this distance, the more novel the sampled genome compared to genomes already in the sample. As expected, the novelty of sampled genomes is strictly decreasing during the sampling process (Fig. 2A, top left). SCARAP allows the user to set the number of genomes to sample, or to set an ANI threshold at which to stop sampling.

To test whether the sampling can be made even faster, we also ran the sampling process using only a subset of 100 core genes. With this reduced core gene set, it took 12 min and 20 s to sample all genomes, or 1.03 s per genome. This increased speed came at the cost of some loss in resolution, whereas the cANI values based on the full core genome yielded 43 genomes with a novelty of zero (i.e. genomes whose core gene sequences are identical to those of genomes already sampled), the cANI values based on 100 core genes (cANIsub values) led to 248 genomes (34.4%) with a novelty of zero (Fig. 2A, top right, points at the very bottom of the plot). This reveals that the use of a smaller number of core genes comes at the cost of reduced resolution: very closely related genomes can no longer be distinguished.

The cANIsub should be strongly correlated to the full cANI in order to be useful. Therefore, we assessed the correlation between the cANI and cANIsub by computing both measures for a set of independent genome comparisons. The result showed a clear correlation between the two, but it was not as strong as expected (Fig. 2B, left side; Spearman correlation of 93.1%). We hypothesized that this lack of a stronger correlation was caused by ‘outlier genes’: genes with a much lower gene-specific ANI than most of the other core genes. Such outlier genes would have a strong impact on the cANI value but would not necessarily be sampled for the cANIsub computation.

To test our outlier gene hypothesis, we computed a trimmed version of the cANI measure (tcANI), where 5% of the genes with the lowest and highest ANI values are removed before cANI computation, effectively removing any outlier genes that are present. For the tcANI, the correlation between its value calculated from the full core genome and from 100 core genes was much stronger (Fig. 2B, right side; Spearman correlation of 97.9%). This confirmed our hypothesis that outlier genes have a strong impact on the classical ANI measure and that the tcANI is a way to reduce this impact. We therefore recommend the use of this trimming procedure in ANI calculations, with the only disadvantage being a slight reduction in resolution (Fig. 2A, bottom left and right).

The strength of the sample module is that it can be used for very rapid sampling of a (small) subset of the total number of genomes. To illustrate this sampling process in phylogenetic terms, we plotted the sampling order of the first 10 sampled genomes of *Lactiplantibacillus* next to a phylogenomic tree of these genomes. In a phylogenetically representative sampling process, older ancestors (internal nodes) are discovered before more recent ancestors. For our sampling, this was the case for eight out of the nine “ancestor discoveries” implied by the sampling order of the first ten genomes (Supplementary Fig. S4). This shows that the sample module can be used to obtain a sample of genomes that is close to a phylogenetically representative sample, without the need to actually construct a phylogeny.

### 3.4 Application of SCARAP on a large dataset of *Lactobacillales* genomes

The SCARAP pan module was benchmarked on a relatively small dataset to allow other pangenome tools to finish in a realistic timeframe. However, the main aim of SCARAP is to allow pangenome analysis for datasets that are taxonomically diverse but also very large. To demonstrate how the SCARAP modules can be combined to process such datasets, we applied the core, sample, and pan modules to infer a representative pangenome for over 31 000 genomes of the order *Lactobacillales*. First, we downloaded all 31 801 high-quality genome assemblies that represent strains of *Lactobacillales* according to the Genome Taxonomy Database (Parks *et al.* 2018), belonging to 1 040 different species. Next, we extracted 100 core genes from these assemblies and used them to representatively subsample a maximum of 100 genomes per species. In the subsampling process, we also applied a threshold of maximum 99.99% tcANI between sampled genomes, ensuring no two sampled genomes were more similar than this threshold. The result was a set of 6 715 representative genomes. Finally, we inferred the pangenome of the subsampled genome dataset. The resulting pangenome consisted of 93 512 orthogroups.

**Figure 3.**
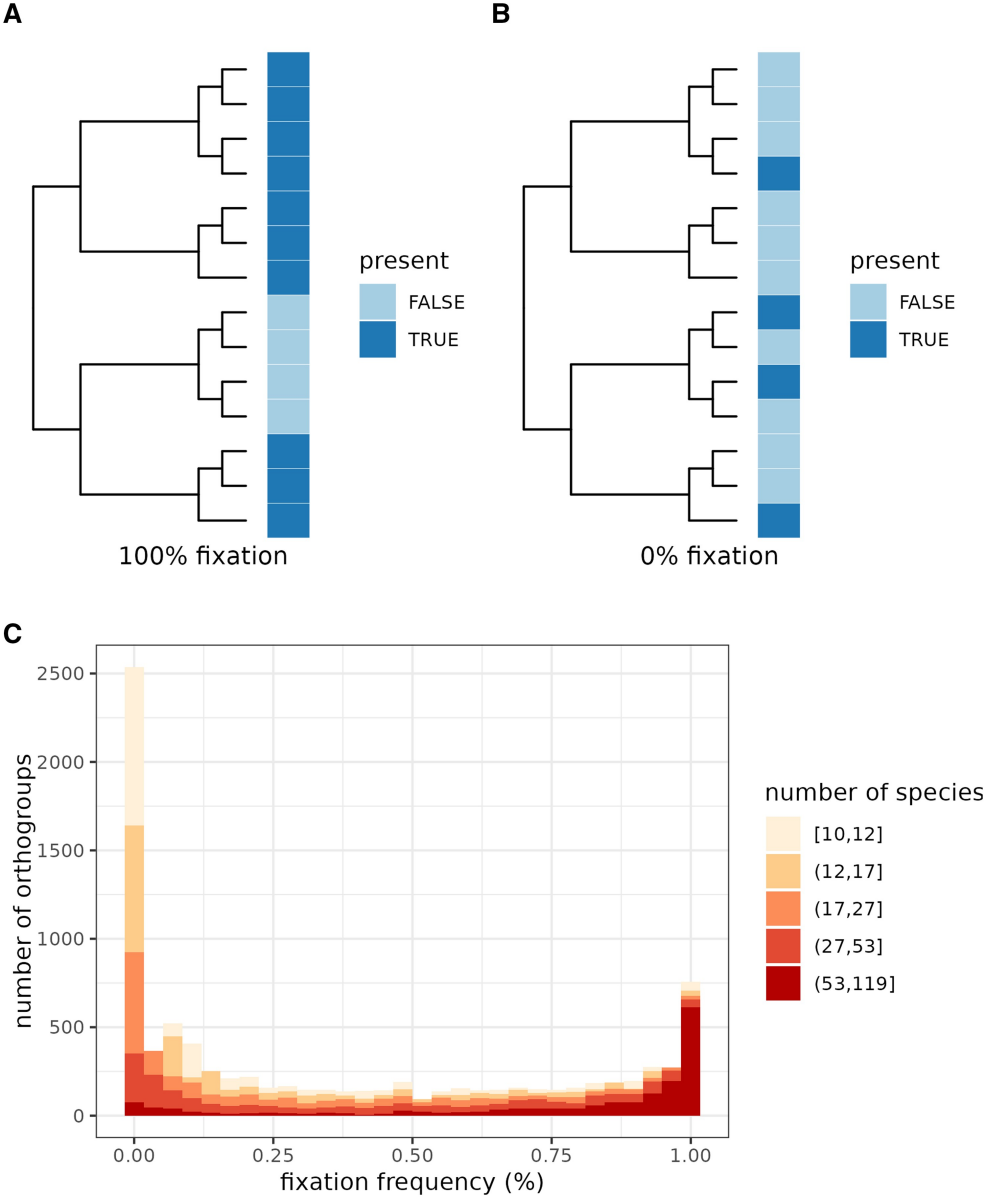
Exploration of the pangenome of *Lactobacillales*. (A) Schematic illustration of an orthogroup with a fixation frequency of 100%. (B) Schematic illustration of an orthogroup with a fixation frequency of 0%. (C) Histogram of fixation frequencies. Fill color represents the number of species where an orthogroup is present in at least one genome.

Our *Lactobacillales* pangenome contained genomes from different species, and in many cases multiple genomes belonging to the same species. This feature of the dataset allows the exploration of some unique aspects of pangenome structure. One illustration is an investigation of the orthogroup fixation frequency, which we define as the proportion of species where the orthogroup is part of the species-level core genome relative to the number of species where the orthogroup is part of the species-level pangenome (i.e. present in at least one genome of the species). A fixation frequency of 100% indicates that an orthogroup is ‘core’ in all species where it is present (Fig. 3A and B). However, it does not necessarily mean that the orthogroup is present in all of the species; only that it is either part of the core genome of a species or fully absent.

To study the fixation frequency of *Lactobacillales* orthogroups, we first subsetted the pangenome to the 119 species with at least 10 genomes available and to the 8903 orthogroups present in the pangenome of at least 10 such species. We then calculated the fixation frequency of each orthogroup. This analysis revealed a bimodal distribution of the fixation frequency of orthogroups. On one end of the distribution, 11.4% of orthogroups had a fixation frequency of at least 95% (Fig. 3C). This is not surprising; these are the genes that are conserved because of their essential function. However, 32.6% of orthogroups had a fixation frequency lower than 5%. This is striking, given that we considered only orthogroups present in at least 10 species.

We see three possible explanations for why so many orthogroups would have a low fixation frequency. A first explanation could be frequent between-species horizontal gene transfer. This process would generate an abundance of recently gained genes within genomes that have not yet had the time to be purged from or fixated in their species. Second, many orthogroups could be under negative frequency-dependent selection, meaning that they confer a larger fitness advantage when they are rare (Levin *et al.* 1988). A known example is restriction–modification systems that confer immunity against phages; the rarer the system, the smaller the chance that it encounters phages that have evolved to evade the system. Finally, orthogroups could be part of the machinery of mobile elements like prophages and conjugative elements. Such genes spread because of the transfer mechanisms they encode, but there is no selection pressure for them to be retained in their prokaryotic host and thus become part of the core genome.

We hypothesized that the most extreme examples of low-fixation-frequency orthogroups would encode mobility machinery components. To test our hypothesis, we selected the top 10 orthogroups in the *Lactobacillales* pangenome that had a fixation frequency below 5% and were present in the largest number of species (Supplementary Table S1). We determined the potential functions of these orthogroups by searching them on the InterPro database (Paysan-Lafosse *et al.* 2023) using representative sequences. For seven of these orthogroups, we found a match with an InterPro gene family. Six of these were gene families known to be bacteriophage specific, providing some support for the idea that the most extreme nonfixating orthogroups are related to mobile genetic elements.

## 4 Conclusions

In this work, we demonstrated that the SCARAP toolkit enables rapid core- and pangenome analysis of large, multi-species prokaryotic datasets with reliable results. In addition, we illustrated that the toolkit can be leveraged to gain novel evolutionary insights from multi-species datasets. Specifically, an exploration of the fixation frequency of *Lactobacillales* orthogroups revealed a surprisingly large number of orthogroups with a fixation frequency close to zero, with the most extreme cases often being associated with phage-related functions.

## Supplementary Material

btae735_Supplementary_Data
