# Supplementary material for: SCARAP: scalable cross-species comparative genomics of prokaryotes
Source: Bioinformatics. 2024 Dec 11;41(1):btae735. doi: 10.1093/bioinformatics/btae735 (PMC11681940; doi:10.1093/bioinformatics/btae735)
Supplement: btae735_Supplementary_Data [file btae735_supplementary_data.zip › supplementary-text-1.pdf]

The pangenome module of SCARAP

Fast initial clustering with MMseqs2

The SCARAP pan pipeline consists of two main stages: (1) fast clustering with MMseqs2 and (2) iterative cluster splitting (Figure 1). In the fast clustering stage, preclusters are first created by running the cascaded clustering workflow of MMseqs2 (`cluster` module) with a minimum sequence identity of 20% and a minimum bidirectional coverage of 50%. The clustering itself is performed with the greedy set cover algorithm, which selects sequences with the highest number of matches to other sequences as cluster centers. In order to increase sensitivity, the preclusters are then clustered into "superclusters" with a profile-based strategy, also using MMseqs2. First, a profile is created for each precluster with the `result2profile` module and the profiles are converted to consensus sequences using the `profile2consensus` module. Then, the consensus sequences are compared against the profiles with the `search` module, with an e-value cutoff of 0.001 and a minimum target coverage of 50%. Finally, the results of this comparison are used as input for a clustering step with the `clust` module, using the greedy incremental clustering algorithm. This creates the superclusters that will be iteratively split in the second stage of our pipeline.

Iterative cluster splitting adds flexibility

In stage II of our pipeline, superclusters are iteratively split into the final gene families (or orthogroups). Per iteration, a split of the cluster into two subclusters is proposed and the proposed split is assessed by a stopping criterion based on genome overlap between the subclusters. If the split is accepted, the splitting process continues for both subclusters. If the split is rejected, the cluster is final and becomes an orthogroup. To propose a split, the sequences are first aligned with MAFFT. A pairwise sequence identity matrix is then constructed and converted to a distance matrix by subtracting all values from one. Next, average-linkage hierarchical clustering is performed based on this distance matrix. Finally, the clustering tree is split into its two top-level clusters.

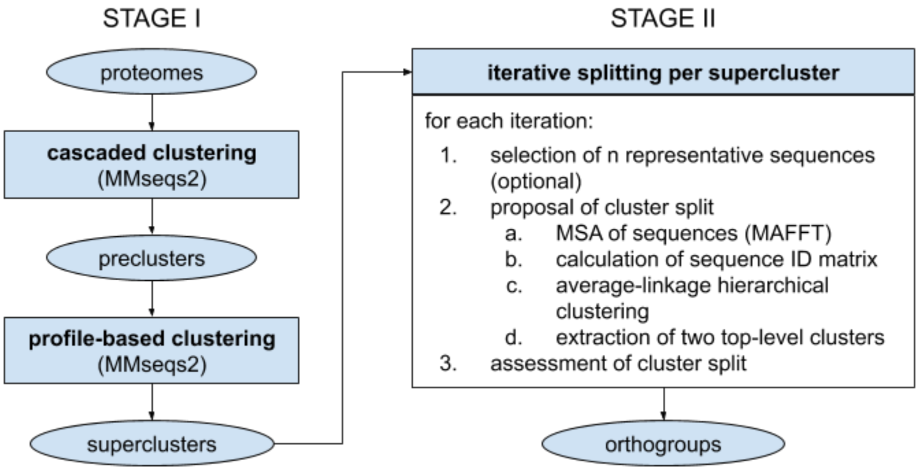

Figure 1: **Overview of the SCARAP pangenome pipeline.**

The assessment of a proposed split is based on the overlap that is observed between the genomes represented in the first and second subclusters. Formally, we define the observed genome overlap (OGO) as:

$$OGO = \frac{|G1 \cap G2|}{min(|G1|, |G2|)} \quad (1)$$

Where G1 is the set of distinct genomes represented in subcluster 1 and G2 is the set of distinct genomes represented in subcluster 2.

To see how the OGO can inform us on whether a cluster should be split or not, we consider its two most extreme cases. In one extreme, the two proposed subclusters are never present in the same genome; the OGO is equal to zero. We interpret this as a sign that the subclusters are functionally redundant (i.e., no neo- or subfunctionalization occurred). In this case, the split is rejected; the subclusters stay together and form a single orthogroup. In the opposite extreme, the two proposed subclusters are both present in at least one copy in all genomes, corresponding to an OGO of one. This is an indication that the subclusters diverged in function. The split is accepted, and the splitting process will then continue for both subclusters.

In most cases, the OGO will not be zero or one. To assess intermediate scenarios, the OGO is compared to the percentage of overlapping genomes that can be expected under a null model where the genes of the cluster are assigned to random genomes. We call this the expected genome overlap (EGO). If the OGO is greater than or equal to the EGO, the split is accepted and the splitting process continues; if the OGO is smaller, the split is rejected and the splitting

process stops. We now derive a formula for the calculation of the EGO for a given proposed split.

When N genes of a (sub)cluster belong to genome i, we say that the frequency of genome i in the (sub)cluster is N. Under the null model, we assume that the frequencies with which genomes are observed follow a multinomial distribution with the same per-genome observance probabilities for the complete cluster, subcluster 1 and subcluster 2:

$$\vec{N_c} \sim mult(n_c, \vec{p})$$

$$\vec{N_{s1}} \sim mult(n_{s1}, \vec{p})$$
$$\vec{N_{s2}} \sim mult(n_{s2}, \vec{p})$$

Where $\vec{N_c}$, $\vec{N_{s1}}$ and $\vec{N_{s2}}$ are the genome frequencies in the complete cluster, subcluster 1 and subcluster 2 (respectively); n_c, n_{s1} and n_{s2} are the total number of genes in the complete cluster, subcluster 1 and subcluster 2; and $\vec{p}$ are the genome observance probabilities.

We further assume that the genome observance probabilities are simply equal to the observed relative frequencies of the genomes in the complete cluster:

$$p_i = n_i/n_c$$

Where n_i is the number of times a gene of genome i was observed in the complete cluster.

It was shown by Emigh (1983) that under these assumptions, the expected number of distinct genomes (more generally, the number of distinct classes or NDC) present in a (sub)cluster can be calculated as:

$$NDC(n, \vec{p}) = K - \sum_{i=1}^{K}(1 - p_i)^n \quad (2)$$

Where K is the total number of distinct genomes.

We can now formally define the EGO and derive a formula for its calculation in practice. Based on Equation 1, we define the EGO as:

$$EGO = \frac{|G1' \cap G2'|}{min(|G1'|, |G2'|)}$$

Where G1' and G2' are now not the observed counts of distinct genomes, but the expected counts. This can be reworked to:

$$EGO = \frac{|G1' \cup G2'| - |G1'| - |G2'|}{min(|G1'|, |G2'|)}$$

Or:

$$EGO = \frac{NDC(n_c, \vec{p}) - NDC(n_{s1}, \vec{p}) - NDC(n_{s2}, \vec{p})}{min(NDC(n_{s1}, \vec{p}), NDC(n_{s2}, \vec{p}))} \quad (3)$$

Which can be calculated with the help of Equation 2.

Representative gene selection adds scalability

Our supercluster splitting strategy based on multiple sequence alignment and hierarchical clustering of all genes is relatively fast for smaller superclusters but scales poorly to larger superclusters (and thus also to larger genome datasets). To make the splitting strategy scalable, we designed a strategy to select representative sequences for a cluster in linear time. This strategy consists of linear-time subclustering into N "linclusters" (a term we will use to avoid confusion with super- or subclusters; see Figure 2), followed by the selection of the median-length sequence per lincluster as representative. We perform this representative sequence selection for each cluster with more than M sequences. The proposal of a binary cluster split is then performed with the representative sequences only, which drastically speeds up the process, especially for large clusters. For the assessment of the proposed split, the representatives are reinflated with all members of their lincluster. The default values for N and M are 32 and 512, respectively.

Our linear-time sequence clustering algorithm, which we call FICLIN (for FIxed number of Clusters in LINear time), is a modified version of the heuristic GMM algorithm described by Ravi et al. (1994). To cluster a set of sequences into N groups, the first step is the selection of N "seed sequences" in such a way that the seeds are as diverse as possible. More specifically, we look for the subset of seed sequences whose maximum pairwise sequence identity is as small as possible. The seed selection is initiated by selecting the longest sequence as the first seed and aligning it against all sequences with the `align` module of MMseqs2. The resulting pairwise sequence identities of the seed against the sequences are stored in a "seed matrix". Then, seeds are added one by one until the desired number of seeds (clusters) is reached. New seeds are selected as follows. The seed matrix is first used to calculate the maximum sequence

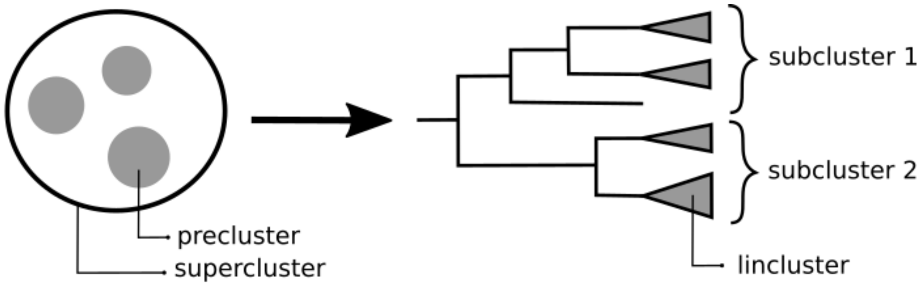

Figure 2: **Schematic overview of the terms used for different types of clusters.**

identity of each sequence to the seeds already in the selection (MAXID). Next, the sequence with the smallest MAXID is selected as the next seed; this is the sequence that is furthest removed from the current set of seeds. If multiple sequences are tied for the smallest MAXID, the longest one is selected. The selected seed is then aligned against all sequences, and the sequence identity values are added to the seed matrix. When the desired number of seeds has been reached, the clustering process is completed by assigning each sequence to the seed to which it shows the highest sequence identity.

References

Emigh, T. H. (1983). On the number of observed classes from a multinomial distribution. *Biometrics*, 39(2):485–491.

Ravi, S. S., Rosenkrantz, D. J., and Tayi, G. K. (1994). Heuristic and special case algorithms for dispersion problems. *Operations Research*, 42(2):299–310.
