# Supplementary material for: SCARAP: scalable cross-species comparative genomics of prokaryotes
Source: Bioinformatics. 2024 Dec 11;41(1):btae735. doi: 10.1093/bioinformatics/btae735 (PMC11681940; doi:10.1093/bioinformatics/btae735)
Supplement: btae735_Supplementary_Data [file btae735_supplementary_data.zip › supplementary-text-2.pdf]

Maximum likelihood estimation of orthogroup prevalence

In situations where a genome dataset includes genomes with a lower completeness (e.g. metagenome-assembled genomes or MAGs), determining the core genome using a fixed prevalence threshold may be challenging because the observed prevalence of the genes is likely lower than their true prevalence. To alleviate this issue, we developed a method that jointly estimates the true prevalence values of all genes as well as the completeness values of all genomes given their pangenome in the form of a gene presence/absence matrix. The procedure assumes a simple model where the probability of observing a gene i in a genome j is the product of the prevalence of the gene in the species (r_i) and the completeness of the genome (c_j):

$$p(s_{ij} = 1) = r_i c_j$$

The likelihood function of the full gene presence/absence matrix in a set of genomes then becomes:

$$L = \prod_i \prod_j \left[(r_i c_j)^{s_{ij}} (1 - r_i c_j)^{1 - s_{ij}} \right]$$

Which gives a log-likelihood of:

$$l = \sum_i \sum_j \left[s_{ij} log(r_i c_j) + (1 - s_{ij}) log(1 - r_i c_j) \right]$$

The algorithm maximizes this log-likelihood in an iterative way. It first initializes all genome completeness values to one, and then alternates between optimizing the gene prevalence values given the current genome completeness values and optimizing the genome completeness values given the current gene prevalence values. This alternation goes on until the likelihood stops increasing.

The likelihood maximizations for an individual prevalence or completeness value are performed by the `optimize` function in R, which numerically optimizes a function for a single parameter, on the interval $[0, 1]$.

We used this algorithm to determine the gene prevalence values (and genome completeness values) for each species with ten or more genomes available. Genes with an estimated prevalence of 80% or more in their species were considered core.
