# Supplementary material for: SCARAP: scalable cross-species comparative genomics of prokaryotes
Source: Bioinformatics. 2024 Dec 11;41(1):btae735. doi: 10.1093/bioinformatics/btae735 (PMC11681940; doi:10.1093/bioinformatics/btae735)
Supplement: btae735_Supplementary_Data [file btae735_supplementary_data.zip › supplementary_figures_tables[AU].pdf]

Supplementary figures and tables

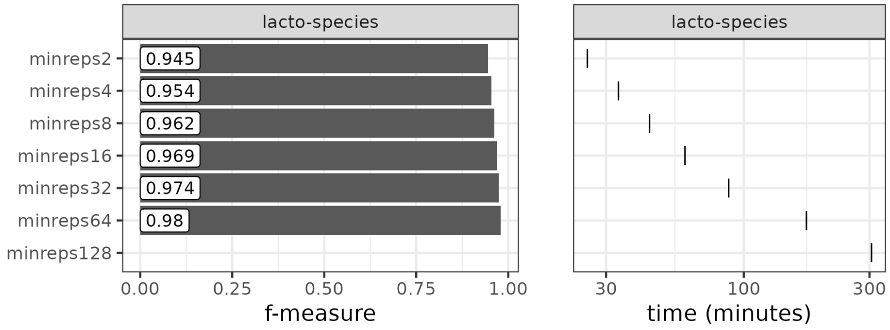

Figure 1: **Optimization of the `pan` `min-reps` parameter.** When splitting larger gene clusters, the SCARAP `pan` module selects representative sequences to align instead of aligning all sequences. The minimum number of representative sequences to use is controlled by the `min-reps` parameter. The default value for this parameter was optimized by running `pan` with a very high value for `min-reps` (128) and then comparing lower `min-reps` settings to these results. Left: F-measure for various `min-reps` settings compared to `min-reps = 128`. Right: Speed for the various `min-reps` settings.

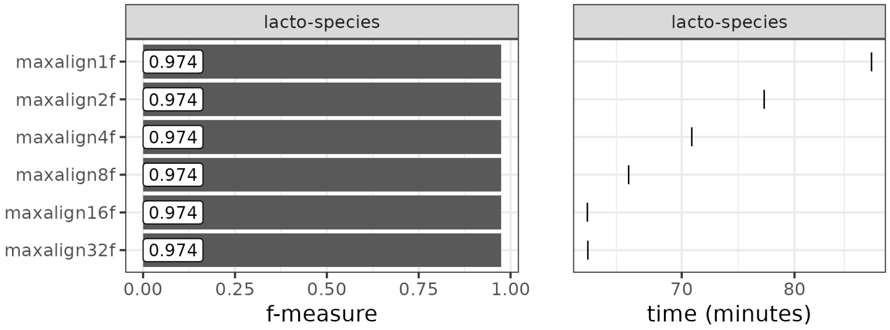

Figure 2: **Optimization of the `pan` `max-align` parameter.** The maximum number of sequences to align in the cluster splitting process of the SCARAP `pan` module is controlled by the `max-align` parameter. For cluster sizes below this value, representative sequences are selected and aligned instead of all sequences. This parameter was optimized by running SCARAP `pan` with various multiples of the `min-reps` parameter as `max-align` values, for `min-reps = 32`. Left: F-measure for SCARAP `pan` runs with different `max-align` values. E.g., "maxalign32f" refers to a `max-align` value of 32 x 32, or 1024. Right: speed of the SCARAP `pan` runs.

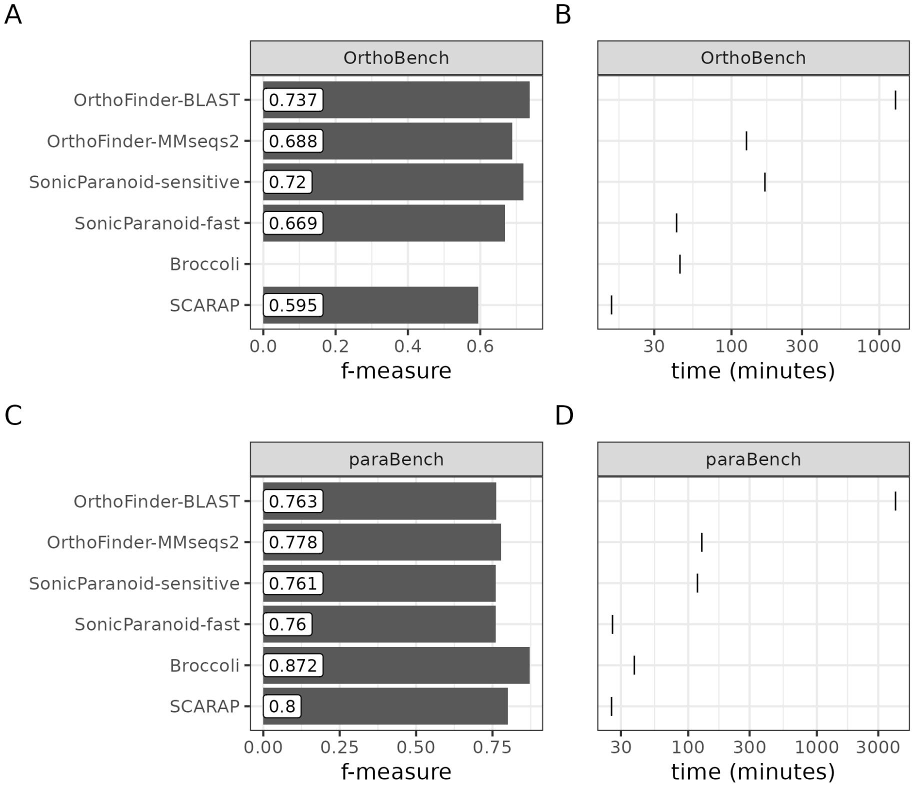

Figure 3: **Benchmarks of the pan module on eukaryotic datasets.** A) F-measure of pangenome tools on the OrthoBench dataset of eukaryotic genomes. B) Running time of pangenome tools on the OrthoBench dataset. C) F-measure of pangenome tools on the paraBench dataset of eukaryotic genomes. D) Running time of pangenome tools on the paraBench dataset. The F-value of Broccoli for the OrthoBench dataset could not be calculated because it assigns some genes to more than one orthogroup.

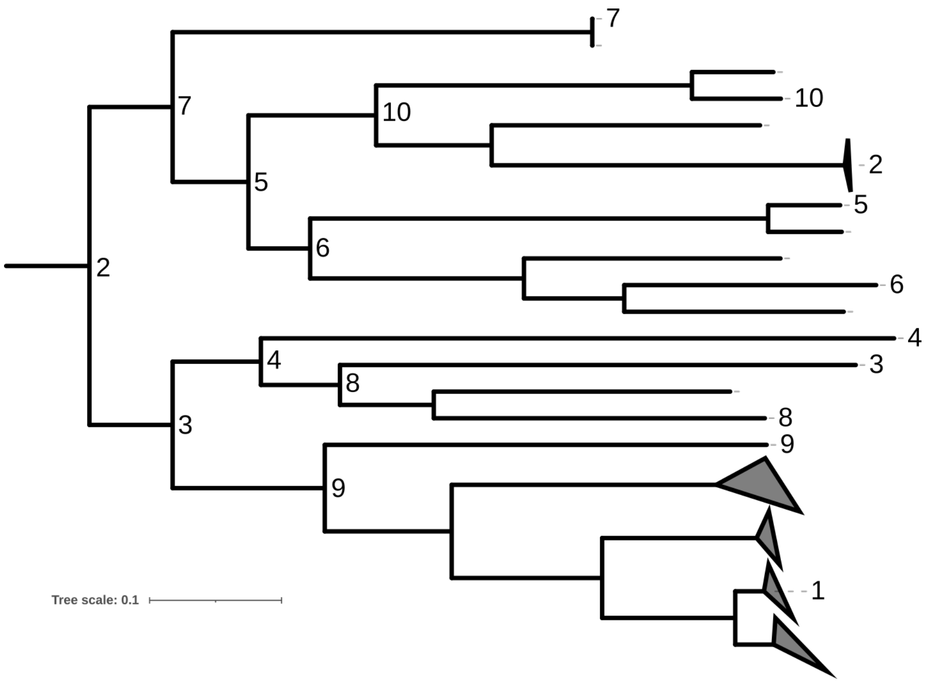

Figure 4: **Phylogenetically representative sampling with the SCARAP `sample` module.** Core genome phylogeny of 720 genomes belonging to 19 species of the genus *Lactiplantibacillus*, where clades consisting of same-species genomes were collapsed. The first ten sampled genomes by the SCARAP `sample` module are indicated in their sampling order, along with discovery of ancestors (internal nodes) that their sampling implies. Tree was visualized with iTol (`https://itol.embl.de/`).

Table 1: **Top ten systematically accessory orthogroups.**

**orthogroup**	**pan-genomes where present**	**accessory genomes where present**	**interpro family**	**function**	**mobility-associated protein**
F12174_16	110	105	IPR001907	ATP-dependent Clp protease proteolytic subunit	no
F17686_1	104	99	IPR021145	Portal protein	phage-related
F01153_1	96	94	IPR005335	Terminase small subunit	phage-related
F26185_1	93	91	IPR009636	Capsid assembly scaffolding protein	phage-related
F28067_4	93	89	IPR006724	Phage tail tube protein 1	phage-related
F46716_1	93	93	none	unknown	unknown
F00661_1	88	84	none	unknown	unknown
F12858_1	88	88	IPR006432	Portal protein, putative, A118-type	phage-related
F61680_3	87	87	IPR009660	Bacteriophage A500, Gp15	phage-related
F01556_1	86	84	none	unknown	unknown
